# Perovskite Manganites: An Overview of Synthesis, Classification, Characterization, and Applications

**DOI:** 10.3390/ijms27135709

**Published:** 2026-06-24

**Authors:** Marzhan Nurbekova, Mukhametkali Mataev, Moldir Abdraimova, Zhanar Tursyn, Zhadyra Durmenbayeva, Zamira Sarsenbaeva

**Affiliations:** Department of Chemistry, Faculty of Natural Sciences, Kazakh National Women’s Teacher Training University, Gogol, 114/1, Almaty 050000, Kazakhstan; nurbekova.m@qyzpu.edu.kz (M.N.); durmenbayeva.zh@qyzpu.edu.kz (Z.D.)

**Keywords:** perovskite manganites, synthesis methods, crystal structure, oxygen nonstoichiometry, magnetic properties, electrocatalysis

## Abstract

Perovskite manganites (AMnO_3_) and perovskite-like manganites (A′_1−x_A_x_MnO_3_) are complex oxide materials that have attracted significant attention from the scientific community in recent years due to their structural flexibility, mixed-valence state, tunable electronic configuration, and multifunctional properties. This review systematically analyzes the synthesis methods, structural classification, and physicochemical characterization of perovskite manganites, as well as their magnetic, optical, electrical, dielectric, and catalytic properties. The influence of solid-state reactions, sol–gel, Pechini, hydrothermal, co-precipitation, microwave, and other mild chemical approaches on phase purity, morphology, particle size, and oxygen stoichiometry was examined. The structural diversity of perovskite and perovskite-like manganites, including simple ABO_3_, double perovskites, multilayer, and low-dimensional systems, was characterized in relation to their functional properties. The review discussed the capabilities of methods for synthesizing and analyzing morphological properties, demonstrating the role of doping, cation substitution, oxygen vacancies, and Jahn–Teller distortions in controlling material properties. Prospects for the application of perovskite manganites in spintronics, magnetocaloric cooling, photocatalysis, gas-sensing devices, and energy conversion and storage systems were analyzed. This review highlights the structure–property–application relationship in perovskite manganites.

## 1. Introduction

Perovskite manganites are compounds of the form of an ideal ABO_3_ or R_1−x_A_x_MnO_3_, where R1 and A are various atoms, which form a three-dimensional MnO_6_ octahedral lattice; the crystal symmetry of such compounds is maintained within the limits of the Goldschmidt tolerance factor [1,2] and the electronic and magnetic properties are determined by the bond geometry between Mn–O–Mn. Perovskite-like manganites are those that arise from a perovskite structure but are layered (Ruddlesden-Popper type: 2D perovskites—A_n+1_B_n_Mn_3n+1_ [3], bilayer manganites—(R,A)_n+1_Mn_n_O_3n+1_ [4]) or structural distortion phases, i.e., phases with distorted symmetry and stoichiometry. In studies, this difference and structural change are considered important in analyzing the structure–property relationship, as octahedral rotations, layer stacking, and bond defects (oxygen-deficient phase) concentration directly affects electronic correlation, magnetic ordering, and the transport mechanism [5,6].

Perovskite manganites are a significant class of multifunctional complex oxide materials characterized by structural diversity and unique physicochemical properties. These materials exhibit large-scale magnetic resistance, metal–insulator transitions, charging and orbital adjustment, and complex magnetic interactions due to bulk lattice interactions, charge, and degrees of freedom of lattice walls associated with the Mn-O skeleton, with variations in many properties [7,8,9]. Perovskites are split into one-dimensional (1D) [10], two-dimensional (2D) [7] and three-dimensional (3D) nanostructures due to the morphology and spatial constraints of manganites. At the nanoscale, perovskite-like manganites exhibit different properties compared to bulk materials. These materials alter electron transport; surface activity increases and varies depending on the degree of magnetic regulation. Structure allows for the regulation of the material’s electronic and magnetic properties, which affects crystal lattice defects and surface energy. One-dimensional structures, such as nanowires, nanorods, and nanotubes, have a large surface area and enable directed electron transport. Owing to these properties, they are considered suitable materials for use in microelectronics, magnetic, and spintronic devices [11].

The terms and features of the synthesis method have a direct impact on the composition, structure, and properties of perovskite-type manganite. The solid-state reaction method is a traditional way of producing such ceramic materials. However, this method has a number of weaknesses. These weak points include: the salts and oxides used for synthesis undergo repeated grinding and heating prior to calcination [12]. The disadvantages of this process include product heterogeneity, the presence of structural defects that inhibit luminescence, the introduction of chemical impurities during repeated grinding and heating, and the presence of particles in the coarse-grained phase. These factors render the resulting material unsuitable for use in coatings [13]. To eliminate these drawbacks and obtain a purer material, a number of new and modified synthesis methods were devised [14]. These approaches include: co-precipitation, solid-phase reactions, hydrothermal synthesis, the Pecini method, gas-phase synthesis, the sol–gel method, green synthesis [5], low-temperature combustion, microwave synthesis, PVD methods (laser ablation, molecular beam epitaxy), and aqueous chemical methods [6].

This review studies the relationship between defect chemistry (oxygen vacancies, cation substitution, and structural distortions) in perovskite manganites and their synthesis methods, as well as their impact on functional properties. Particular attention is paid to identifying synthesis–defect–structure–property correlations and determining the key descriptors for enhancing the electrocatalytic (HER/OER) efficiency of perovskite manganites.

## 2. Classification of Perovskite Manganites

### 2.1. Perovskites

Currently, the term “perovskites” is a broad group of natural and synthesis oxides with a cubic or orthorhombic structure and the general formula ABO_3_, where A and B are metal ions. The name first appeared in connection with calcium titanate (CaTiO_3_), discovered by Rose in the Ural Mountains in 1839; it was later named “perovskite” in honor of the mineralogist L. A. Perovsky. Compounds similar to this mineral include strontium titanate SrTiO_3_ (discovered in 1982 as the rare mineral “tausonite”) and barium titanate BaTiO_3_, which is very rarely found in nature in its pure form. Similar to them (Mg, Fe), there are also silicate perovskites with the formula SiO_3_. Iron and magnesium silicates are found in large quantities in the lower layer of the Earth’s mantle and exist in a stable state under conditions of high pressure and high temperatures. Pure MgSiO_3_ has been found on Earth only in the Tenham meteorite and was named “bridgmanite” in 2014. The name was given in honor of P. W. Bridgman, who received the Nobel Prize in Physics in 1946 [15].

**Figure 1:**
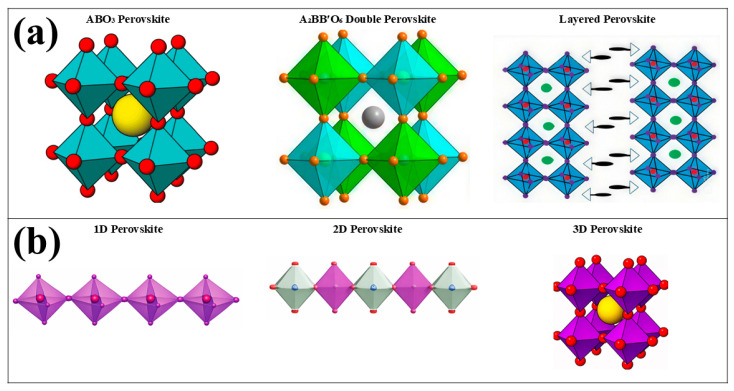
Structural types of perovskites: (**a**) ABO_3_ perovskite, A_2_BB′O_6_ double perovskite, layered perovskite, and low-dimensional perovskite structures; (**b**) 1D, 2D, and 3D perovskite frameworks. The central large sphere represents the A-site cation. B-site cation is located at the center of the BO_6_ octahedron and is not visible in the schematic representation. The smaller surrounding spheres correspond to oxygen atoms.

Perovskites are a group of materials that have a particular crystal structure, named according to the mineral perovskite (CaTiO_3_). Its general chemical formula is written as ABX_3_, where A is a large atom with a 12-coordination, and B is a cation in an octahedral structure formed by oxygen anions. The A-site can be substituted by twenty-seven differently sized elements, and the B-site can be exchanged for thirty-five different elements. Structurally, the A cations are located at the corners of the cube, the B cations are at the center of the cube, and the X anions are on the circumference of the cube, forming a BO_6_ octahedral framework around the B ions [16]. This structure comprises a cubic lattice, whereby the cation at site A is sizeable and occupies the space between the cations at the octahedral site B (Figure 1). Oxygen ions bond cations A and B, maintaining overall cubic symmetry under ideal conditions. These control the redox activity of the perovskite, helping to maximize yield and lower reaction conditions [17].

**Figure 2:**
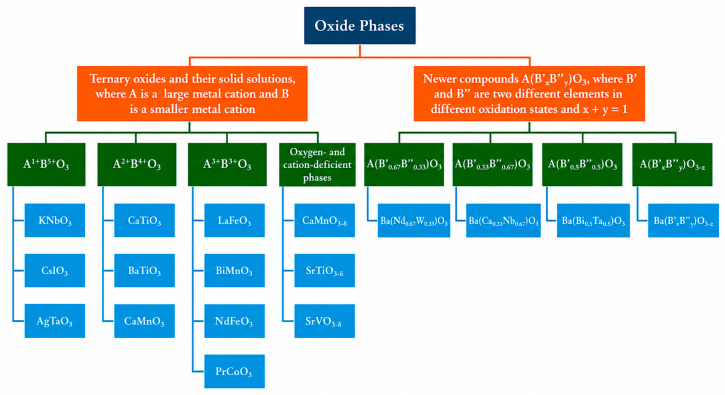
Types of triple oxides [7].

One of the main advantages of perovskites is likely their structural adaptability and stability at room temperature. Furthermore, perovskites are considered an environmentally safe and viable alternative to expensive rare-earth materials such as gadolinium. By substituting ions at the A- and B-sites in the perovskite structure, it has been possible to optimize both the magnetic and thermal properties, which enhances their magnetocaloric effect and improves their efficiency in refrigeration technology [18]. The off-stoichiometric properties of LaMO_3_ compounds (M = Cr, Mn, Fe, Co, Ni) were first described in the work of Nakamura et al. [19]. Perovskites of lanthanum manganite contain trivalent lanthanides and divalent alkaline earth metals in the A-site, whereas the B-site consists mainly of first-row transition metals [20] (Figure 2). 

Perovskite inorganic oxides are high-potential nanomaterials frequently used in electrochemical analysis [21], catalysis and fuel cells [22,23]. Perovskites synthesized at the nanoscale exhibit high catalytic activity, high electronic conductivity, Otto ion coagulation, and high thermochemical plasticity. Therefore, they are used as effective catalysts [24] in important electrochemical processes, such as oxygen reduction [25] and hydrogen production reactions [26]. Perovskite manganites are structured forms of manganites that exhibit a perovskite structure. These materials possess distinct chemical and physical properties that manifest at the nano- or microscale, making them a subject of considerable interest in scientific research [27,28]. Subsequently, such properties were also observed in La_1−x_Sr_x_MO_3_ systems [25,26,27,28].

Perovskite-type manganites constitute a complex group of oxides with the general formula Ln_1−x_A_x_MnO_3_. Ln refers to rare earth elements (La, Pr, Nd, etc.), and A refers to alkali, transition, or alkaline earth elements. In these compounds, Mn ions occupy the B-site in an octahedral center, forming the MnO_6_ structural unit. As the degree of ion exchange and doping in the perovskite structure changes, the symmetry and chemical–physical properties of the crystal lattice change noticeably [29]. LaMnO_3_ in its pure form has an orthorhombic structure (space group Pnma). Doping is a method of altering the structural properties of perovskite by substituting metal ions for manganese in specified proportions (quantities) of the R_1−x_M_x_MnO_3_ compound. Throughout this process, changes in the crystal lattice structure and modifications in the physical and chemical (magnetic, catalytic, optical, electrical) properties are studied based on replacing the metal at the A-site with rare earth, alkali, or alkaline earth metals, and the metal at the B-site with transition metals in the target compositions [28,30]. Jahn–Teller (JT) distortion is clearly observed in this compound, since the e_g_-orbitals of the Mn^3+^ ions are fully occupied. This distortion reduces the Mn–O–Mn bond angles and increases the lattice anisotropy. Therefore, LaMnO_3_ is an insulating material [31,32,33,34]. Furthermore, if rare-earth or transition metal ions are introduced instead of La^3+^ ions (increasing the doping level X), Mn^3+^ ions undergo redox transitions to Mn^4+/2+^ within the system. This change in valence weakens the Jahn–Teller distortion because the e orbital of the Mn^4+^ ion is vacant. Under its influence, a transition occurs from the orthorhombic phase to the rhombohedral (R$\bar{3}$c) phase, and then to the cubic (Pm$\bar{3}$M) structure with increased lattice symmetry. For example, for La_1−x_Me_x_MnO_3_ (Me = metal ion), the lattice parameters changed [35]. The values indicate a reduction in lattice distortion and an increase in structural stability. Phase transitions are noted as the temperature rises: at low temperatures, the orthorhombic phase predominates; at 600–800 °C, it transitions to the rhombohedral phase; and at higher temperatures, to the cubic phase. This phenomenon occurs due to increased ionic vibrations in the oxide crystal lattice and the formation of oxygen vacancies. The introduction of Fe^3+^ ions in compounds such as LaFeO_3_ or FeMnO_3_ also affects the lattice parameters. Since the radius of the Fe^3+^ ion is smaller than that of Mn^3+^, the lattice becomes slightly more compact, and the Mn-O-Mn angle decreases from approximately 160° to 155°. Although this reduces conductivity, it increases structural stability and catalytic activity. Likewise, in perovskite manganites, as the degree of doping increases and the temperature rises, the lattice symmetry increases (Pnma → R$\bar{3}$c → Pm$\bar{3}$m); Mn-O-Mn angle increases and octahedral distortion decreases; the Mn^3+^/Mn^4+^ ion relationship changes and electrical conductivity increases; ferromagnetic properties are enhanced [36,37].

Perovskite manganites exhibit exceptional magnetic, catalytic, and electrocatalytic properties, while halide perovskites are widely used in optoelectronics due to their superior optical properties. Surface chemistry and defect control, however, play a crucial role in both systems. Passivation of surface defects in halide perovskites enhances material stability and light-emitting efficiency, whereas in oxide manganites, control of oxygen vacancies and surface-active sites determines catalytic activity and stability [17,38,39].

### 2.2. Perovskite Manganites

Perovskite manganites are nano- or microstructured forms of manganites that exhibit a perovskite structure. These materials possess distinct chemical and physical properties that manifest at the nano- or microscale, making them a subject of considerable interest in scientific research [27,28].

**Figure 3:**
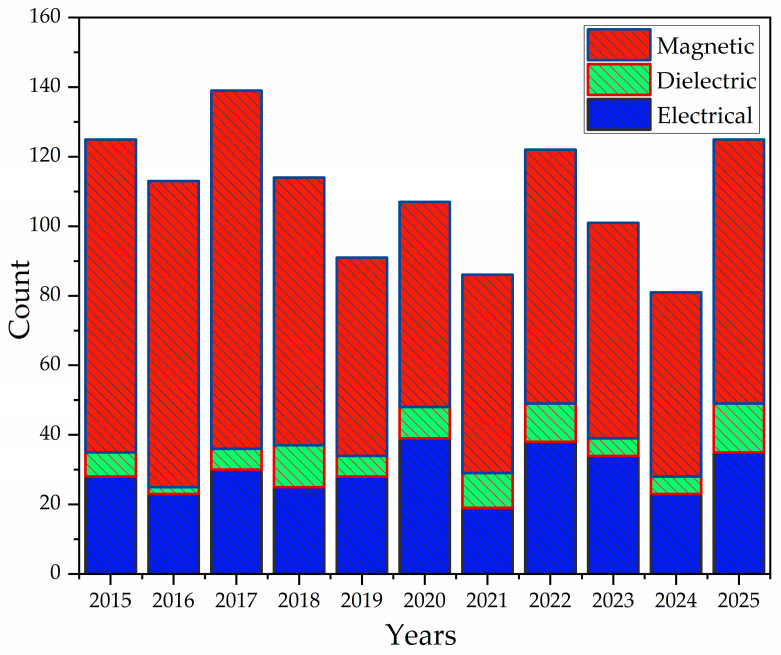
Trends in publications on perovskite manganites highlighting electrical, dielectric, and magnetic properties (2015–2025).

The graph (Figure 3) shows the number of scientific papers devoted to the study of the electrical, dielectric, and magnetic properties of perovskite manganites from 2015 to 2025. The results show that the proportion of studies on magnetic properties is the highest, while the number of publications devoted to electrical and dielectric properties is relatively small. The increase in the number of studies across all areas over the years demonstrates the growing importance of perovskite manganites in science and technology as functional materials. The graph shows the change in the number of scientific publications on the electrical, dielectric, and magnetic properties of perovskite manganites from 2015 to 2025. The bibliometric data were taken from the Scopus database. The search was conducted using the following keywords: “perovskite manganites” and “electrical properties,” “perovskite manganites” and “dielectric properties,” as well as “perovskite manganites” and “magnetic properties.” The search was performed by document title, abstract, and keywords, and the results were grouped according to publication year (2026).

## 3. Synthesis Methods of Manganites

The synthesis method directly determines the phase purity, crystallite size, morphology, oxygen stoichiometry, and functional properties (magnetic, electrical conductivity, electrocatalytic) of the finished material. The specialization of the method is crucial for the synthesis of perovskite manganites in nanoform [5,40].

**Figure 4:**
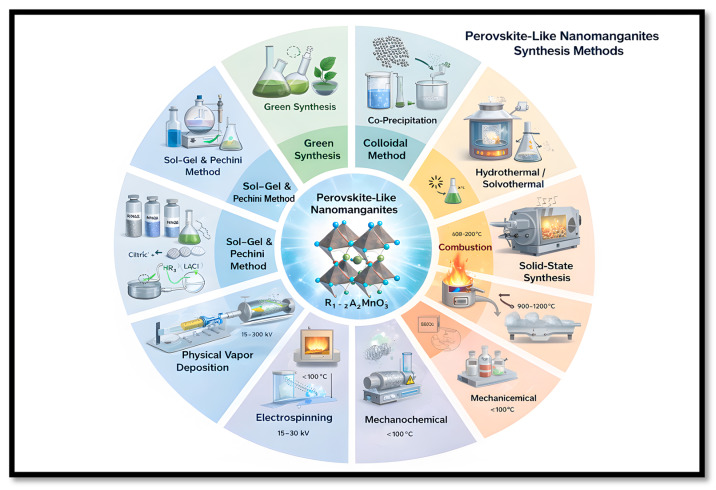
Classification of synthesis methods for ABO_3_ and A_1–x_A′_x_MnO_3_ perovskite manganites.

Shown in Figure 4 classification of synthesis methods for perovskite-type oxides corresponding to the formulas ABO_3_ and A_1−x_B_x_O_3_. It presents the main technologies widely used for producing these materials. These synthesis approaches allow for control over the crystal structure, particle size, morphology, and structural defects of the resulting materials, which in turn determine their electrical, magnetic, and catalytic properties.

An effective method for synthesizing perovskite-structured manganites is the sol–gel method. This method enables the preparation of dispersive and phase-pure oxide materials based on homogeneously mixed precursors at low temperatures, at the molecular level [41]. The sol–gel method is built on forming a complex between metal nitrates or acetates and organic complexing agents (such as citric acid, ethylene glycol, or polyols). Initially, solutions of metal ions are prepared and mingled in a stoichiometric composition. The citric acid is then added to the mixture, forming a citrate complex. Polyether reaction occurs when ethylene glycol is introduced into the formed solution, resulting in a viscous sol. While heating and evaporating, the sol gradually transforms into a gel. The gel is dried at a temperature of 120–200 °C, ensuring complete removal of organic components. The dried powder is further calcined in air or in an oxygen atmosphere in the range of 600–900 °C. At this stage, the organometallic complexes decompose, and a perovskite phase forms with Me(A)–O and Me(B)–O bonds. The calcination time and temperature directly influence the crystal structure, particle size, and physicochemical properties of the resulting manganites. Manganites obtained by the sol–gel method are characterized by a finely dispersed, porous structure and a high surface area. Such materials exhibit high activity in electrocatalytic, magnetic, and photocatalytic processes. The main advantages of the method are the homogeneous mixing of the starting reagents, phase purity, low calcination temperature, and the ability to control the morphology of the resulting material [42,43,44]. Perovskite manganites demonstrate a close relationship between synthesis conditions, defect structure, Mn valence states, and functional properties. Parameters like calcination temperature, annealing atmosphere, precursor ratio, and synthesis method significantly influence the crystal structure, grain size, oxygen non-stoichiometry, and the Mn^3+/^Mn^4+^ ratio [45,46].

Several methods exist for synthesizing perovskite manganites. Table 1 shows that, along with traditional solid-state synthesis, the sol-gel method, the Pecini method, hydrothermal synthesis, and other modern methods can be applied to produce materials with high uniformity and a nanostructure.

The Pechini method (polymerizable complex method) is a low-temperature, high-yield method for synthesizing multicomponent oxides and perovskite-like compounds. The essence of this approach lies in the formation of a homogeneous organic–inorganic polymer gel through the complexation of metal cations using citric acid (C_6_H_8_O_7_) and polymerization with ethylene glycol (C_2_H_6_O_2_). As a result of subsequent thermal treatment of the gel, the organic component burns out, and highly dispersed metal oxide powders are formed. For the synthesis of 3D manganites using the Pecini method, solutions of metal salts (nitrates) are typically used. First, these salts are dissolved in distilled water. The molar ratio of citric acid to the total amount of metal cations is chosen to be approximately 2:1 or 3:1. This allows the citric acid to fully complex all the cations. In the next step, ethylene glycol is added. The molar ratio of citric acid to ethylene glycol is approximately 1:2–1:4. The mixture is continuously stirred at a temperature of 80–120 °C, resulting in the formation of a viscous polymer solution (resin). This resin is dried at 120 °C, ensuring the evaporation of organic components. The dried sample is pre-calcined at a temperature of 250–400 °C. At this stage, the organic residues are completely decomposed, yielding an amorphous precursor of metal oxides. Final calcination is carried out for 2–6 h at a temperature of 700–800 °C. This process results in the formation of a perovskite-type manganite crystalline phase. Advantages of the method include the mixing of all cations at the molecular level, the possibility of low-temperature synthesis, and the high reactivity of the resulting product. Disadvantages include the need to ensure complete removal of organic waste and sensitivity to the calcination conditions. 

## 4. Properties of Perovskite Manganites

### 4.1. Structural, Physical, and Functional Properties

**Figure 5:**
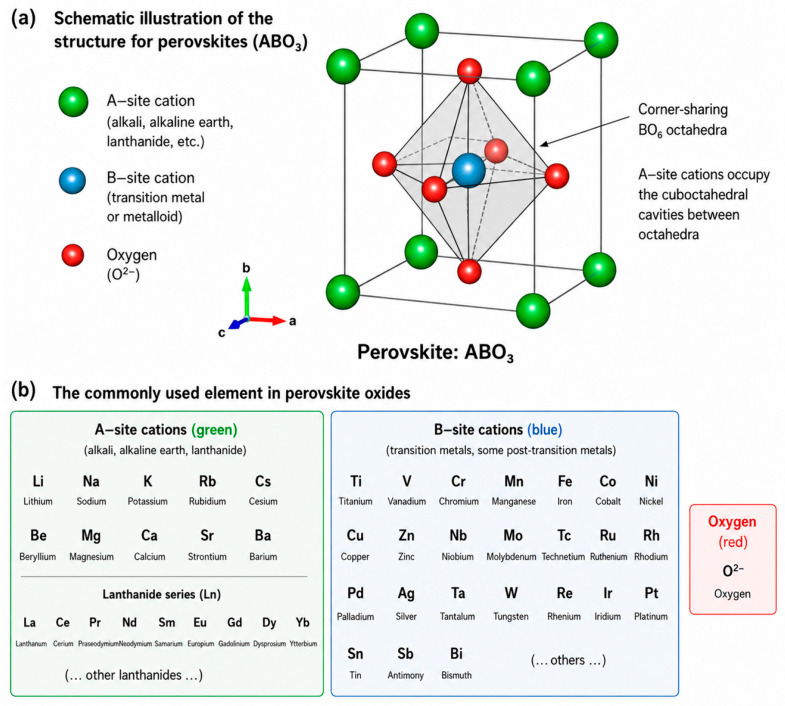
Structure of a perovskite oxide and typical cation distribution. (**a**) Schematic illustration of the crystal structure of perovskite oxides (ABO_3_), showing BO_6_ octahedra with cations at the corner sites, located at cuboctahedral sites. (**b**) Commonly used elements in perovskite oxides, where Group A cations are shown in green, Group B cations in blue, and oxygen in red.

Physical properties of nanostructured perovskite manganites encompass a broad range of structural, magnetic, electrical, optical, and mechanical characteristics. Their properties depend on the material’s chemical composition, the ionic radius of the cations, and the synthesis conditions [47,70]. Perovskite manganites have the potential to crystallize in differing crystalline symmetries depending on the radius of the cation in the A-site and the processing conditions. The most common structures include hexagonal (space groups P6ccm, P6./mmc) [47,71], orthorhombic (Pbnm) [72] and rhombohedral (R3c, R3m) [73,74] structures. In [47], structured perovskite manganites (RMnO_3_, R = Y, Er, Yb) have an average crystal size of approximately 68–72 nm. One of the reasons for the particle size dependence may be rare earth ionic radii, but this parameter is also affected by synthesis conditions, crystallization characteristics, and particle aggregation. Jahn–Teller oscillations and the tilt of MnO_6_ octahedra affect structural stability and electronic transitions; structural distortion increases as the cation radius at the A-site decreases (Figure 5).

Physics of manganites with mixed valence is characterized by complex interactions between spin, charge, and lattice degrees of liberty. Fundamental to understanding transport mechanisms are models that treat the metal–dielectric transition as the result of competition between pair exchange and carrier localization effects. Specifically, charge carriers in the paramagnetic region have been shown to form small polaron pairs, whose motion is driven by thermally activated hopping [75,76]. Approaches based on the concept of a mobility threshold shift under disordered conditions successfully describe the giant magnetoresistance effect as a result of changes in activation energy under the influence of temperature or a magnetic field [77]. Studies of high-quality single crystals confirm that near T_C_, temperature and field dependencies of the resistivity are determined by a change in the activation energy, depending linearly on the square of the magnetization [78].

Perovskite manganites are notable for their complex magnetic, electrical, optical, and mechanical properties. SrMnO_3_ and BaMnO_3_ are antiferromagnetic insulators, and their Curie temperatures range from 233 to 280 K; doping can lead to the formation of a ferromagnetic state via a double-exchange mechanism between Mn^3+^ and Mn^4+^ ions Mn^3+^/Mn^4+^ [71,74]. The materials exhibit the Colossal Magnetic Resistance (CMR) effect and are promising for sensor devices due to their high temperature coefficient of resistance (TCR). Hexagonal manganites have a bandgap of approximately 1.3–1.64 eV and exhibit ferroelectric properties at high temperatures. Furthermore, their mechanical properties depend on the crystal structure: hexagonal phases are typically brittle, while orthorhombic phases are relatively ductile [47,71,73,74,79].

The interaction of the octahedra in this three-dimensional lattice determines the material’s magnetic interactions and electronic properties. Perovskite structure often deviates from ideal cubic symmetry and transitions into tetragonal, orthorhombic, or rhombohedral phases. Such fluctuations depend on ion radii, the degree of doping, and synthesis parameters. Structural distortions influence the overlap of orbitals, altering properties such as the Curie temperature (T_c_), magnetization strength, and magnetocaloric effect. In ferromagnetic mixed perovskite manganites Ln_1−x_M_x_MnO_3_ (where Ln are rare-earth elements, often La, Pr, Nd, Sm, and M—alkaline earth elements: Sr, Ca, Ba), the KT electronegativity in the metal–semiconductor transition temperature (TMI) region increases markedly under the influence of an external magnetic field (H) [80]. The thermochemical cycle of perovskite involves its gradual reduction at high temperature or low oxygen pressures. Reduction occurs as follows:

ABO_3_ → ABO_3−δ_ + δ2O_2_ (1)

where δ signifies the non-stoichiometric degree within the perovskite structure.

The structure of manganite perovskite depends largely on interactions arising from differences in the sizes of its constituent ions, electronic instability (Jahn–Teller effect), the degree of doping, and changes in the crystal lattice. The symmetry of the material, its magnetic ordering, and electron phases are determined by these factors. The factor that determines the structural strength of classic perovskite is the index that shows the ratio of the radii of the cations at sites A and B to their oxygen ion radius [37,81,82]. In manganites containing Mn^3+^ (t_2_g3 eg1), the Jahn–Teller effect plays a vital role as an electronic factor: it aligns the Mn^3+^ octahedron along a single axis, eliminating orbital degeneracy. This causes isotropy in bond lengths, with all Mn-O bonds—short, medium, and long—coexisting side by side (e.g., 1.927 Å, 1.929 Å, and 2.07 Å in LCMCO). JT distortion leads to long-range orbital ordering, such as the alternation of dz2 orbital direction in the a-B plane [82,83]. Goldschmidt Tolerance Factor (t) is the essential criterion for structural stability in perovskites; it compares the cation and oxygen ion radii at the A-site and B-site. Values of t = 1 indicate an ideal cubic structure, whereas lower values cause the MnO_6_ octahedra to rearrange and block each other to fill the space as a result of differences in ion sizes. The double-doped manganite La_0.7_Ca_0.3_Mn_0.6_Co_0.4_O_3_ (LCMCO) with t = 0.832 confirms a distorted orthorhombic phase. Bi-component perovskite fills the A and B-sites in Mn_2_O_3_, with Mn ions at the A-site that are smaller in the larger 12-coordinated cavity, creating strong octahedral packing reaching 15–18° [84]. Octahedral twist and rotation (Glazer notation) of MnO_6_ octahedra is a key factor in symmetry changes in perovskite manganites [85]. Glazer notation is utilized to describe octahedral deformations (a^−^b^+^a^−^ or a^0^a^0^c^−^) where the magnitude and direction of rotation (in phase or out of phase) are identified relative to the pseudocubic axes. The rotation of the octahedra causes the Mn-O-Mn bond angle to deviate from the ideal value of 180°, which, in turn, narrows the width (W) of the electronic band. The width of the band decreases, the mobility of charge carriers decreases, and the electrical resistance in the system increases. It leads to the stabilization of the insulating and antiferromagnetic phases, rather than the metallic and ferromagnetic states. In the Pr_0_._5_Sr_0_ Prmno compound, local Torus distortions arise due to the difference in the radii of Pr^3+^ and Sr^2+^ ions. These distortions create internal stress fields, facilitating phase transitions between the paramagnetic insulating state and the ferromagnetic metallic state in the material [38,82]. Recent research (LaMn_1−x_Ga_x_O_3_ (LMGO)) has shown that shear strain (Γ_5_^+^) may be the primary factor driving the transition from an orthorhombic structure to a pseudocubic phase. The system establishes a trilinear coupling term (Γ^5+^M^2+^M^3+^) between shear strain, octahedral phase rotation (M_2_^+^.), and Jahn–Teller orbital coupling (M_3_^+^). It is important to note that shear strain cannot only be a consequence of orbital disorder but also its cause. When the crystal lattice is shear-stable, long-range orbital regulation could be suppressed even in the presence of a large number of active Mn^3+^ Jahn–Teller ions [83]. Doping affects the perovskite structure in two ways: it alters the lattice geometry and regulates the concentration of charge carriers. Doping at the A-site (insertion of Ca^2+^ or Sr^2+^ instead of La^3+^) alters the Mn^3+^/Mn^2+^ ratio, modifying ferromagnetism and electrical conductivity via a double-exchange mechanism (double exchange). Doping at the B-site (e.g., introducing Co, Ga, or Fe instead of Mn) stabilizes the known oxidation states, suppressing JT distortions and altering exchange interactions. In the LaMnO_3_ system, Co doping leads to lattice contraction and stabilizes the Mn^4+^ state, resulting in increased thermal stability and electrical conductivity of the material [82,83].

**Figure 6:**
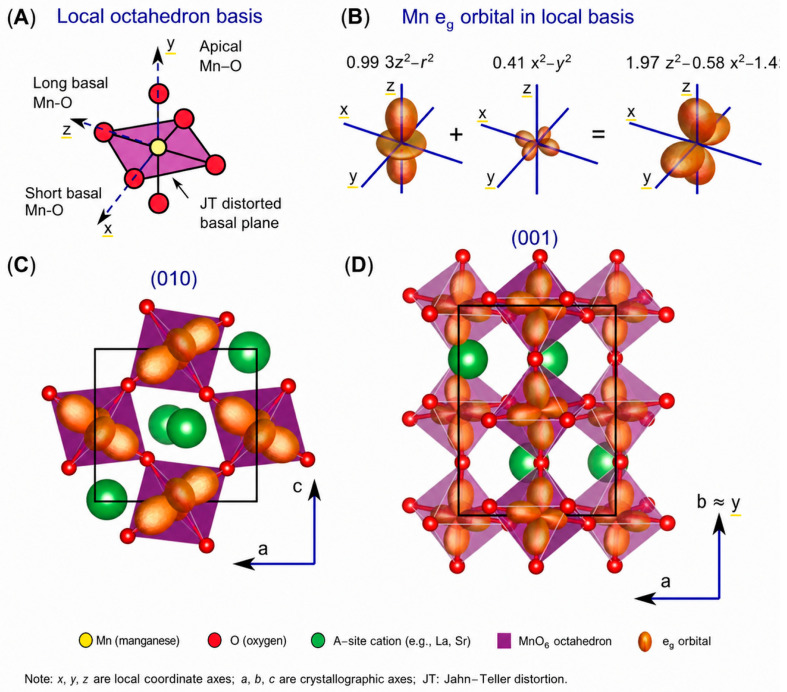
Orbitals in the LaMnO_3_ unit cell from a U|J = 8|2 eV calculation. (**A**) MnO_6_ octahedron with Jahn–Teller distorted plane and local basis vectors labeled. (**B**) Visualization of the occupation of the 3z^2^ − r^2^ and x^2^ − y^2^ states in the rotated basis of the density matrix as well as their superposition for the total local e_g_ occupancy (plotting the occupation times the orbital expressed in spherical harmonics). (**C**) The ordering of the occupied e_g_ shell (1.97z^2^ − 0.58 × 2 − 1.4y^2^) in the (010) Jahn–Teller distorted FM coupled plane. (**D**) The ordering of the occupied e_g_ shell in the (001) plane with AFM coupling along b. Note x, y, z is the local octahedron basis, and a, b, c lattice vectors correspond to x′, y′, z′ global (pre-rotation) calculation basis [85].

Figure 6 illustrates the ordering of orbitals and JT distortion in LaMnO_3_, including the MnO_6_ octahedron; it shows the occupancy of orbitals and the arrangement of occupied orbitals in different crystallographic planes.

Table 2 presents the crystal structures (space groups) of a variety of nanoperovskite manganites, along with the main physical and chemical properties and areas of application. The compounds listed crystallize primarily in rhombohedral (R$\bar{3}$c), orthorhombic (Pnma, Pbnm), hexagonal (P6_3_cm, P6_3_/mmc), cubic (Pm3m, Fm-3m), and monoclinic (P2_1_/n) structures. These materials are distinguished by their magnetic (ferromagnetic, antiferromagnetic), electrical, and dielectric properties, as well as by the interaction between mixed-valent Mn^3+^/Mn^4+^ ions. Through alloying or cation substitution, their Curie or Neel temperatures, magnetization, electrical conductivity, and catalytic activity can be modified. The application of manganites presented in the table in various technologies, such as spintronics, magnetic refrigeration, catalysis, energy storage devices, gas sensors, and photocatalysis, demonstrates that this is a multifunctional material.

### 4.2. Magnetic Properties

Magnetic properties of perovskite manganites are driven by how charge, spin, orbital, and lattice degrees of liberty are linked [103,104,105,106]. The materials are of intense scientific interest due to phenomena such as the colossal magnetoresistance (CMR) and the magnetocaloric effect (MCE) [16,86,107,108]. Magnetic state of manganites is determined by the competition between a pair of complementary indirect exchange mechanisms, mediated primarily by oxygen ions: double exchange (DE) and superexchange (SE). The double exchange mechanism arises from electron transfer between ions of different valences, Mn^2+^ and Mn^4+^, by way of 2P oxygen orbitals, providing ferromagnetic ordering and metallic conductivity [106,109,110]. The superexchange mechanism, on the other hand, occurs between ions of the same valence (Mn^3+^–Mn^3+^ or Mn^4+^–Mn^4+^) [103,111,112,113,114], leading to antiferromagnetic ordering and the formation of a dielectric state. Microwave studies of CMR manganites, particularly those using ferromagnetic resonance (FMR), confirm the close relationship between their microwave properties and their magnetic and transport characteristics. FMR parameters reflect magnetic anisotropy, the degree of homogeneity, and the quality of the samples, and also correlate with their conductivity and low-temperature behavior [115,116].

Magnetic properties of manganites are closely tied to the distortion of the crystal lattice. The JT effect, special to Mn^3+^ ions, causes deformation of the MnO_6_ octahedra, leading to changes in the Mn–O bond length and the Mn–O–Mn angles [16,50,110]. These changes determine the band width (W) and the strength of the magnetic interaction [109,110]. Further, structural stability is assessed using the Goldschmidt tolerance factor (t_G_): its deviation from the ideal value leads to tilting and rotation of the MnO_6_ octahedra, which typically weakens the double exchange mechanism and lowers the Curie temperature (T_C_) [50,103,112,117]. Magnetic properties of manganites can be effectively tuned by replacing ions (doping) at the A- and B-sites. As a result of replacing trivalent ions (La^3+^) with divalent (Ca^2+^, Sr^2+^, Ba^2+^) or monovalent (Na^+^, K^+^, Ag^+^) ions at the A-site, a mixed valence of manganese (Mn^3+^/Mn^4+^) is formed, which promotes the emergence of ferromagnetism [16,109,111,112,118]. In the B-site, the substitution of Mn ions with other transition metals, such as Cr, Co, Ni, Fe, or Cu, often weakens the double exchange mechanism, enhancing the antiferromagnetic as-synthesized structure and lowering T_C_ [16,109,112,119,120].

**Figure 7:**
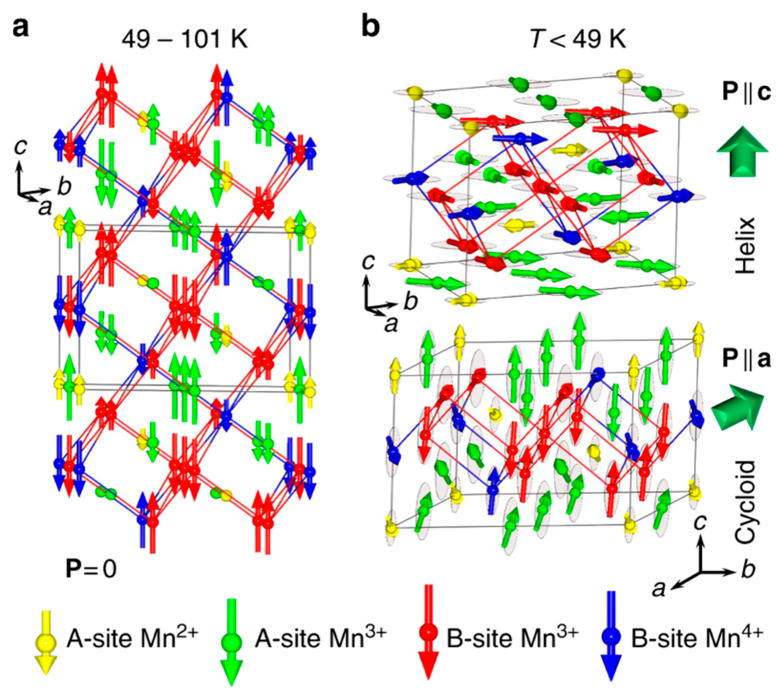
Magnetic structures of the perovskite-type Mn_2_O_3_. (**a**) Schematic representations of the magnetic structure in the commensurate high-temperature phase (49 K < T < 101 K). This structure is a longitudinal spin density wave with the unit cell eight times bigger than the cell of the parent R$\bar{3}$ (2 $\sqrt{{2a}_{p}}$ × 2 $\sqrt{{2a}_{p}}$ × $\sqrt{{3a}_{p}}$) structure. It combines two types of the B-site and three types of the A-site Mn-layers stacked along the c-axis. (**b**) Schematic representations of the magnetic structure in the incommensurate low-temperature phase (T < 49 K). This structure combines both cycloidal and helical components. P refers to the induced electric polarization [83].

Figure 7 shows the change in the temperature-dependent magnetic structure of the Mn_2_O_3_ perovskite compound. In the 49–101 K range, the commensurate structure of the material has a high-temperature phase characterized by magnetic ordering in the form of a longitudinal spin-density wave (SDW). It differs from the basic lattice by having a larger unit cell and a complex overlap of Mn ions in the A- and B-sites. Upon cooling below 49 K, the system transitions to a low-temperature phase, where the magnetic structure consists of a superposition of cycloid and helicoid components. The complex spin configuration leads to a violation of spatial inversion symmetry, inducing an electric polarization (P). It indicates a correlation between the magnetic and electric properties in the Mn_2_O_3_ system, suggesting that it exhibits multiferroic behavior.

**Figure 8:**
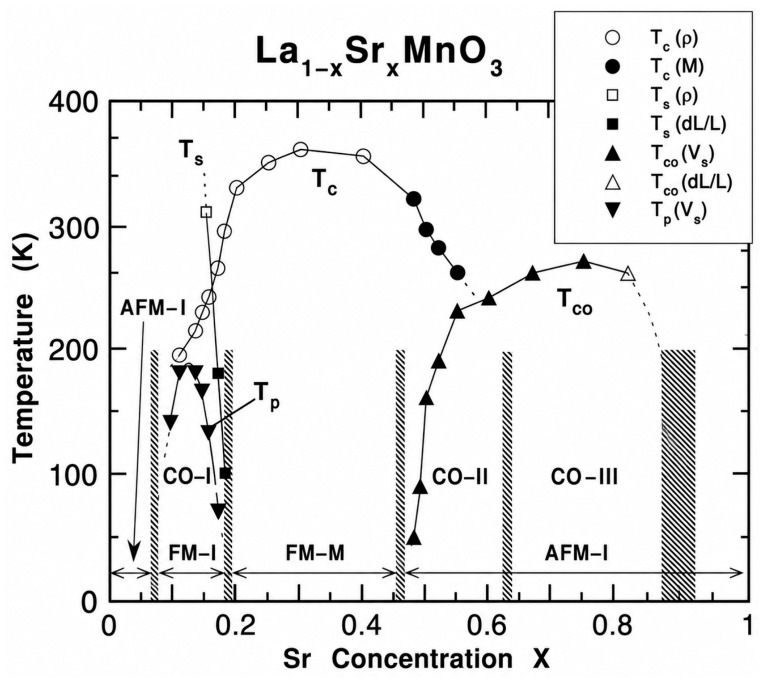
Phase diagram of La_1−x_Sr_x_MnO_3_ as a function of temperature and doping x. AFM-I: antiferromagnetic insulator; FMI: ferromagnetic insulator; FM-M: ferromagnetic metal; CO: charge ordering state; T_C_—Curie temperature; T_N_—Néel temperature; T_P_—polaron ordering temperature; T_S_—structure transition temperature; T_CO_—charge ordering transition temperature [124].

Figure 8 shows the magnetic phase diagram of La_1−x_Sr_x_MnO_3_ compound, which depends on the Sr concentration and temperature. Diagram illustrates the transitions between the ferromagnetic (FM), antiferromagnetic (AFM), and charge–ordered (CO) phases and their corresponding transition temperatures. Hatched regions indicate phase boundaries or concentration intervals for phase transitions.

First-order (FOPT) and second-order (SOPT) magnetic phase transitions are observed in manganites [109,110,112,121]. Second-order transitions are characterized by the absence of thermal and magnetic hysteresis and are considered suitable for use in magnetic cooling technologies [16,105,109,112]. In some compositions, such as La_0.7_Ca_0.3_MnO_3_, a first-order phase transition occurs, leading to a significant change in entropy; however, this phenomenon occurs over narrow temperature intervals [17,106,111]. In nanostructured manganites, magnetic properties are often explained by the “core–shell” model: a ferromagnetic core is surrounded by a chaotic or “magnetically inactive” shell, which leads to a decrease in saturation magnetization [11,122]. The phenomenon of superparamagnetism can be observed in very small particles. Furthermore, manganites are characterized by the simultaneous coexistence (phase separation) of different magnetic phases [111,119,121], whereas the Griffiths phase is characterized by the formation of ferromagnetic clusters in the region above the Curie temperature and anomalous changes in magnetic susceptibility [103,105,123].

**Figure 9:**
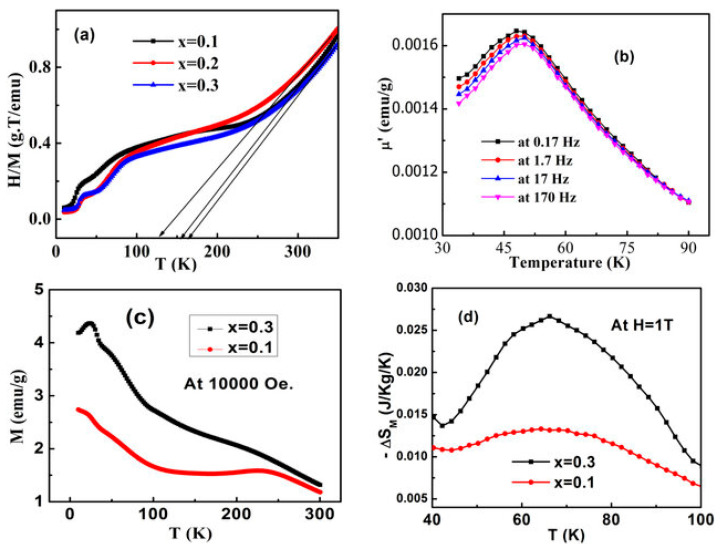
(**a**) Inverse magnetic susceptibility versus temperature curves for Sm_0.5_Ca_0.5–x_Sr_x_MnO_3_, (**b**) ac susceptibility M′(T) curves with frequencies 0.17 Hz, 1.7 Hz, 17 Hz, 170 Hz for the sample with x = 0.3, (**c**) M versus T plots under 10,000 Oe (1 T) magnetic field for the samples with x = 0.1 and x = 0.3 and (**d**) magnetic entropy change (−ΔSm) for x = 0.1 and x = 0.3 under an applied magnetic field H = 10,000 Oe (1 T) [125].

Figure 9 shows the temperature-dependent magnetic properties of the compound Sm_0.5_Ca_0.5−x_Sr_x_MnO_3_. Figure presents curves of magnetic susceptibility, AC susceptibility, magnetization, and changes in magnetic entropy, and also depicts the effect of the Sr mixture on the material’s magnetic and magnetocaloric properties.

**Figure 10:**
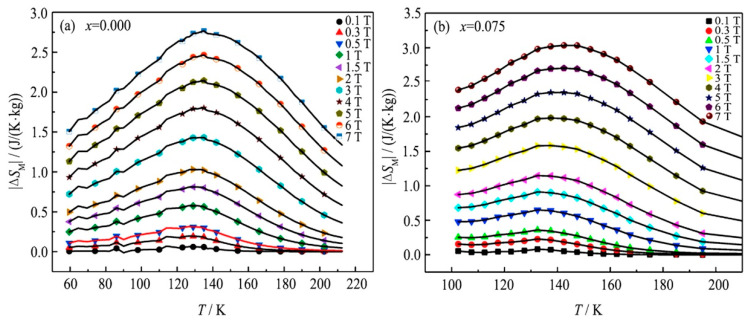
The maximum magnetic entropy change |Δ*S*_M_| value of the samples La_0.9–*x*_Eu*_x_*Sr_0.1_MnO_3_ (*x* = 0.000, 0.075) near Curie temperature (T_C_) reaches 2.76 and 3.03 J/(K·kg), respectively. In addition, the relative cooling power (RCP) is found to be 425.28 and 443.53 J/kg. Both samples have the potential to realize magnetic refrigeration in the high temperature region (T > 77 K) [128].

Figure 10 shows the dependence of the change in magnetic entropy (|ΔS_M_|) on temperature for polycrystalline samples. As the external magnetic field increases, the value of |ΔS_M_| also increases, and its maximum is observed near T_C_. In a 7 T magnetic field, the maximum valori for the two samples are 2.76 and 3.03 J/(K·kg), respectively. Eu^3+^ doping enhances the ferromagnetic double exchange effect and leads to improved magnetocaloric properties. It should be noted that as the magnetic field increases, the maximum shifts to a region of slightly higher temperature. Given that the magnetic properties of perovskite manganites are highly sensitive to external magnetic fields and temperature changes, they can be utilized in various functional devices. Particularly, these materials are considered promising for magnetic refrigerators and spin devices (spin valves and magnetic tunnel junctions) based on the magnetocaloric effect, as well as in temperature sensors and bolometers—that is, sensing systems based on the temperature coefficient of resistance (TCR) [16,57,108,111,112,113,119,126,127].

### 4.3. Optical Properties

Optical properties of perovskite manganites are determined by their electronic structure, the spatial configuration of the crystal lattice, and the interplay of structural and electronic changes resulting from doping [5,129,130]. Such materials possess a wide range of optical properties: from semiconductor states with adjustable bandgaps to semimetallic properties. In addition, perovskite manganites are characterized by intense light absorption bands and pronounced magneto-optical effects [129,130]. Doping has a significant effect on the bandgap energy of perovskite manganites. As the amount of strontium in the Nd_1−x_Sr_x_MnO_3_ system increases, Eg increases from 1.8 eV to 3.2 eV, which is associated with a change in the Mn^3+^/Mn^4+^ ion ratio [129]. Similarly, the introduction of vanadium into La_0.7_Pb_0.3_Mn_1−y_V_y_O_3_ increases the value of Eg [131], while in Nd_2_Ni_1−x_Co_x_MnO_6−δ_ compounds, the effect of cobalt is relatively weak [132]. In contrast, the introduction of copper ions into the Nd_2_Ni_1−x_Cu_x_MnO_6_ system reduces the forbidden band gap [133]. In contrast, Mg-doped rare-earth manganites are characterized by a wide bandgap, whereas RMnO_3_-type hexamanganites (R = Y, Er, Yb) have a relatively narrow Eg value and demonstrate the ability to effectively absorb visible light [5,47].

Nonlinear optical parameters of third-order double perovskite manganites were investigated using experimental methods including the Z-scan technique. In particular, the compound La_2_CoMnO_6_ exhibits a combined manifestation of saturated absorption (SA) and reverse saturated absorption (RSA). Observed RSA indicates that this material has potential for use in optical limiting devices that protect against high-intensity laser radiation. Furthermore, the third-order nonlinear optical susceptibility (χ^3^) for La_2_CoMnO_6_ has been determined to be approximately 4.34 × 10^−6^ esu, demonstrating that this is a promising material for the development of nonlinear optical devices [134].

Photoluminescence spectroscopy is one of the most important methods for studying electronic states associated with defects in manganites. In the PL spectrum of the La_0.67_K_0.33_MnO_3_ compound, the emission band observed in the region around 695 nm is associated with deep defect states in the electronic structure. A decrease in PL intensity indicates efficient separation of photogenerated charge carriers, which is a favorable factor for photocatalytic processes [135]. Moreover, the Urbach energy (E_U_) is used to assess the degree of structural disorder in the material. This parameter describes the density of localized electronic states located near the edges of the bandgap. For manganites doped with a mixture of Mg, the values of E_U_ range from 0.26 to 0.29 eV, indicating the high crystalline quality of the obtained materials and a relatively small number of structural defects [5].

### 4.4. Electrical and Dielectrical Properties

Electrical transport properties of perovskite manganites are primarily attributed to strong electron–phonon interactions and double exchange (DE) mechanisms between Mn^3+^ and Mn^4+^. Charge transport in these materials is characterized by different conductive properties depending on the temperature range [136,137,138] At high temperatures, conductivity is often mediated by the small polaron hopping (SPH) mechanism, in which charge carriers move between localized states of the crystal lattice via a thermally activated hopping process. At low temperatures, conductivity is often explained by the variable-range hopping (VRH) model, particularly the Mott VRH mechanism, in which electrons hop over varying distances between localized states. Therefore, electrical conductivity in manganites is often thermally activated and increases with rising temperature [137,139,140]. In many manganite systems, a metal–semiconductor or metal–insulator phase transition is observed in the region close to the magnetic ordering temperature T_C_ [136,141,142] In some manganites doped with transition metals, such a two-phase transition is observed in the temperature range of 80–400 K [136]. Furthermore, AC conductivity often follows the universal Jonscher law and increases with increasing temperature and frequency [137,139,143,144]. In perovskite-manganite systems, a negative temperature coefficient of resistance (NTCR) is observed, meaning that electrical resistance decreases with increasing temperature [145,146].

Perovskite-type manganites are characterized by very high values of dielectric permittivity (ε′), which exceed 10^3^ and, in some cases, reach 10^6^. Such high dielectric permittivity makes these materials promising candidates for use in high-frequency energy storage devices and capacitive memory elements [136,137,143]. High dielectric response observed in polycrystalline manganites is often attributed to Maxwell–Wagner-type interface polarization [137,140,147,148,149]. It occurs since the material’s structure is electrically inhomogeneous; that is, the relatively highly conductive crystalline structure is interspersed with high-impedance particles [136,137,149,150]. Upon exposure to an external electric field, charge carriers accumulate at grain boundaries, leading to a pronounced polarization effect and a significant increase in the dielectric constant [140,144,149,151].

Dielectric properties of perovskite-type manganites depend on several key factors, among which doping levels and microstructural features play an important role. The introduction of cations at the a-site (e.g., Sr, Ba, Ca, Na, K) or transition metals (Ni, Cr, Fe, Co, Al, W) in place of Mn alters the Mn^3+^/Mn^4+^ ion balance [136,137,143,144,145,147,152]. It affects the intensity of the double-exchange mechanism and the motion of charge carriers, leading to changes in the material’s electrical and dielectric properties. Dopants such as chromium (Cr) were found to reduce overall electrical conductivity by refining the particle structure and increasing the electrical resistance at the boundaries of fine particles [147]. Furthermore, the microstructure of the material significantly influences its dielectric behavior. Particle size and orientation, as well as the density of particle boundaries, influence the charge accumulation process [137,144,147].

### 4.5. Catalytic Properties

Perovskite manganites refer to a group of functional materials that have been extensively studied in the field of heterogeneous catalysis [51,54,89,153]. Catalytic properties of these materials are primarily associated with the structural flexibility of the perovskite crystal lattice, as it is possible to substitute cations with various elements and create oxygen vacancies, which leads to the regulation of the electronic structure and chemical activity of the material. An important feature of perovskite manganites is the presence of a mixed-valent Mn^3+^/Mn^4+^ redox pair [51,90], which serves as an active site in the oxidation reactions of pollutants such as nitrogen oxide (NO) and formaldehyde (HCHO), ensuring the reversibility of redox processes [51,89,153]. Moreover, the double-exchange mechanism, in which electron transfer between Mn^3+^ and Mn^4+^ ions is mediated by oxygen anions, increases the material’s electrical conductivity, contributing to efficient charge transport and high catalytic activity [51].

Catalytic activity of LaMnO_3_ perovskite could be significantly enhanced through alio-valent doping or the introduction of cation vacancies [89]. Replacement in A-site with alkaline earth metals (Mg, Ca, Sr, Ba) or alkali metals (Li, Na, K, Rb, Cs) increases the proportion of Mn^4+^ ions, enhances the formation of oxygen vacancies, and contributes to an increase in the specific surface area [51,54,153]. Introduction of Mg at the A-site of the LaMnO_3_ structure is more effective in the NO oxidation reaction than substitution at the B-site, as it increases the charge imbalance and enhances the formation of surface-active oxygen species [154]. Additionally, the deliberate introduction of a cation deficiency at the A-site (La_1−x_MnO_3_) modulates the electronic structure of the material, reduces the energy required for oxygen vacancy formation, and increases catalytic activity in low-temperature oxidation reactions [89,138] Doping with Ce [155] stabilizes the charge balance, creating a high density of surface defects and activating the Mn^4+^/Mn^3+^ valence transition, which plays a particularly important role in the decomposition of chlorinated volatile organic compounds, such as chlorobenzene.

Perovskite manganites demonstrate high efficiency in various catalytic processes. It is possible for them to partially oxidize NO to NO_2_ in the nitrogen oxide (NO) oxidation reaction, which is important for the efficient progression of subsequent denitrification processes. Furthermore, materials based on lanthanum manganite can catalyze the complete oxidation of formaldehyde (HCHO), causing it to decompose into CO_2_ and H_2_O at moderate temperatures (<100 °C), which is important for removing pollutants from indoor air [153]. Even during soot combustion, manganites are effective catalysts, particularly in systems doped with alkali metals, which accelerate the oxidation of carbonaceous particles formed during fuel combustion [54]. Some manganite nanoparticles (Nd_0.7_Ca_0.3_MnO_3_) also exhibit high photocatalytic activity in the degradation of organic dyes under sunlight [90]. Nevertheless, there are several challenges associated with the use of perovskite manganites: for example, the toxic effect of chlorine during the decomposition of chlorinated volatile organic compounds (CVOCs), the formation of secondary phases during high-level doping, and the formation of carbonates upon exposure to CO_2_, which reduces the material’s long-term stability [51,154].

ABO_3_ perovskite manganites, in which manganese occupies the B-site, are considered effective and relatively inexpensive electrocatalysts in renewable energy systems, including fuel cells and electrolyzers [156,157]. These materials exhibit high activity in the hydrogen evolution reaction (HER), oxygen evolution reaction (OER), and oxygen reduction reaction (ORR). Electrochemical properties of manganites are significantly improved by doping: the introduction of Bi into the La_0.6_Dy_0.1_Sr_0.3_Mn_(1−x)_Bi_x_O_3_ significantly increases HER activity, lowering the reaction onset potential from −1.389 V to −1.036 V and increasing the current density by approximately tenfold, which is attributed to the ability of Bi sites to adsorb water molecules [157]. Catalytic activity of manganites depends on the filling of EG orbitals with Mn ions, the covalence of the metal–oxygen bond, and the presence of oxygen vacancies, which, in turn, allows the reaction mechanism to transition from an adsorption mechanism (AEM) to a mechanism in which the lattice participates in the presence of oxygen (LOM). Further, in some ferromagnetic manganites (La_0.7_Sr_0.2_Ca_0.1_MnO_3_), the application of an external magnetic field can reduce the OER overpotential by approximately 18% and increase the current density by up to 80% [158] while modifications such as Bi doping can reduce electrochemical impedance and improve ionic conductivity and reaction kinetics. Parameters such as overpotential, Tafel slope, current density, electrolyte composition, and long-term catalyst stability are typically used to evaluate electrocatalytic properties [159]. Research shows that high oxygen mobility, an optimum Mn^3+^/Mn^4+^ ratio, and an increased concentration of surface defects contribute to improved charge transfer and catalytic activity in oxygen evolution and reduction reactions.

**Figure 11:**
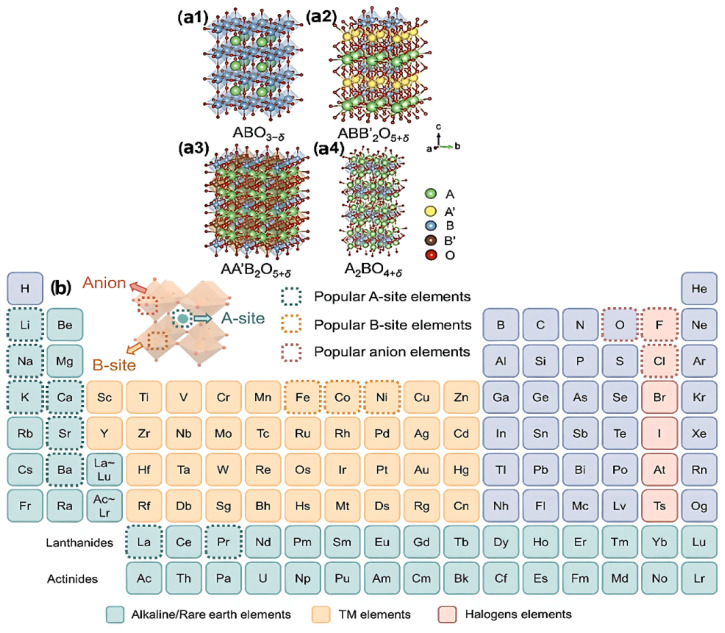
Perovskites as electrode materials for HER. (**a1**) Basic perovskite, (**a2**,**a3**) double perovskites, (**a4**) Ruddleson–Popper phase, (**b**) most common elements in perovskites. Figure is reprinted with copyright permission from the Royal Society of Chemistry [158].

Figure 11 shows the main types of perovskite structures used as HER electrode materials: (a1) classical perovskite (ABO_3−δ_), where A-cations occupy large coordination cavities, and B-cations are located at the centers of BO_6_ octahedra surrounded by oxygen; (a2,a3) double perovskites (ABB′_2_O_5+δ_ and AA′B_2_O_5+δ_); the ordered arrangement of various cations in these structures allows for effective regulation of their electronic and catalytic properties; (a4) the Ruddlesden–Popper layered phase (A_2_BO_4+δ_), which is characterized by the introduction of additional layers between the perovskite layers, improving ion transport and surface reactions. Part (b) shows the elements most commonly found in the perovskite structure: A-positions are typically held by alkali, alkaline earth, and rare earth elements, while B-positions are held by transition metals, allowing their structural and catalytic properties to be tuned over a wide range.

## 5. Application of Manganites

Perovskite manganites are functional materials widely used in various technological fields due to their structural, electronic, and magnetic properties [51]. Applications of these compounds span important areas such as electronics, energy conversion, environmental technologies, and sensor systems [36,50,160]. La_1−x_Sr_x_MnO_3_ (LSMO) manganites are of significant interest in the context of spintronics on account of their stable ferromagnetism and semimetallic properties at room temperature [50,160,161]. They are used in magnetic tunnel junctions, spin valves, and spin-MOSFETs with spin-sensitive metal-oxide semiconductors [97,161]. Further, perovskite manganites play a significant role in high-density magnetic data storage devices, including the read heads of hard disks and magnetoresistive random-access memory (MRAM) systems [57,90,162]. High-quality epitaxial manganite layers enable the creation of oxide quantum devices, ensuring coherent electron transport in quantum wells [161]. Gd_1−x_Ca_x_MnO_3_ and Pr_1−x_MnO_3_ are used in biological neural networks, as a phenomenon where a transition from resistive to conductive states is observed in manganite memristors, and serves as the basis for calculating systems in the development of neuromorphic models, making them promising materials for consideration [162].

The materials in question are widely studied as important functional materials in energy conversion and storage technologies. Strontium-doped lanthanum manganite is considered one of the most effective cathode materials in solid oxide fuel cells (SOFCs) due to its structural stability at high temperatures and mixed ion–electron conductivity [51,163]. Also, since hexagonal rare-earth manganites (h-ReMnO_3_) have a relatively narrow bandgap (about 1.2–1.5 eV), they are considered promising materials for photovoltaic cells, light polarizers, and solar energy conversion systems, and are also used in CO_2_-to-fuel conversion reactions in solar thermochemical processes [40,50]. Moreover, electron-doped SrMnO_3_ and CaMnO_3_ compounds are promising materials for thermoelectric generators that convert high-temperature waste heat into electrical energy [48,51,114,164]. Perovskite manganites also play an important role in energy storage systems: they are used as electrode materials for supercapacitors and batteries (e.g., lithium-ion and lithium-oxygen batteries), while RGO@LSMO-type nanocomposites demonstrate high specific capacity and good cycling stability [165].

Perovskite manganites are widely used as effective functional materials in environmental and catalytic processes. They exhibit high activity as photocatalysts capable of degrading organic pollutants, including azo dyes (methylene blue, malachite green) and certain antibiotics [48,90]. Manganites are effective catalysts in gas-phase catalytic reactions, particularly in methane combustion, hydrocarbon oxidation, and SO_2_ reduction in the presence of CO [51,160]. Owing to their high structural stability, perovskite structures are also considered as a matrix for the immobilization of high-level radioactive actinide residues [160]. Manganites play an integral role in various sensing and thermal technologies: they are used in gas sensors that detect volatile organic compounds such as carbon monoxide (CO), hydrogen sulfide (H_2_S), nitrogen dioxide (NO_2_), ethanol, and acetone [48,160]. Manganites with a high temperature coefficient of resistance (T_CR_) are used in infrared bolometers and uncooled night vision systems; unalloyed CaMnO_3_ is considered a promising material for cryogenic thermal sensors used in extreme conditions, demonstrating a very high TCR value at low temperatures [114,162]. Manganites are also used in magnetic refrigeration systems based on the magnetocaloric effect, a technology that offers an environmentally friendly and energy-efficient alternative to the harmful gases used in traditional vapor-compression refrigeration systems [166].

**Figure 12:**
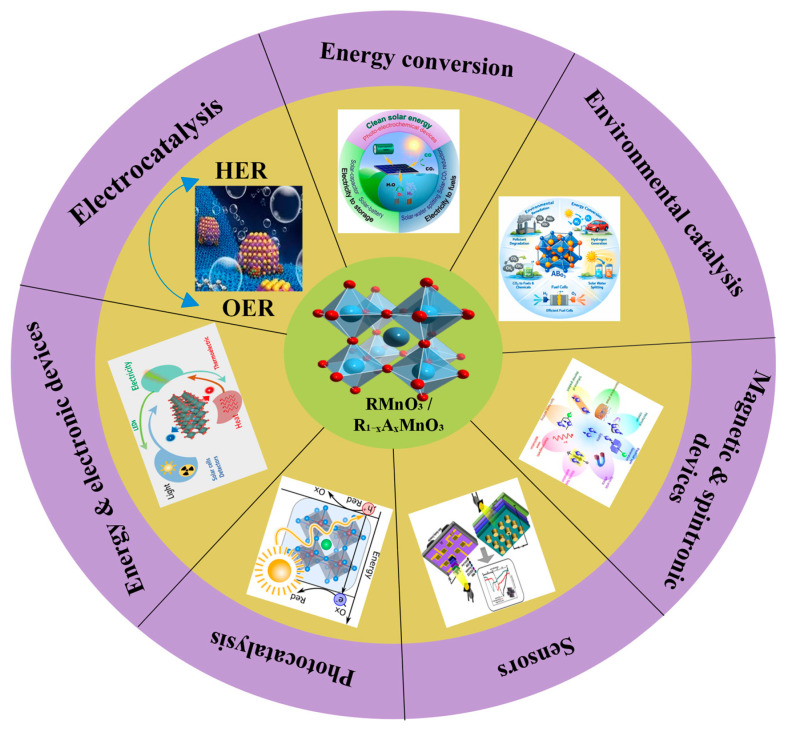
Major application areas of perovskite manganites (RMnO_3_, R_1–x_A_x_MnO_3_).

Figure 12 shows the potential applications of perovskite manganites (RMnO_3_ and R_1−x_A_x_MnO_3_) in various scientific and technological fields. These materials are being extensively studied in energy conversion, photocatalysis, catalysis for environmental remediation, magnetic and spintronic devices, and sensors, as well as energy and electronic devices. Moreover, they play a significant role in electrocatalysis, exhibiting high catalytic activity in processes such as the hydrogen evolution reaction (HER) and the oxygen evolution reaction (OER). The multifunctional properties of perovskite manganites make them promising materials for energy, electronics, and environmental technologies (Figure 12. Author-designed schematic summary of the principal application fields of perovskite manganites, prepared on the basis of the literature reviewed in this work).

Perovskite manganites and perovskite-like manganites constitute a promising group of oxide materials that exhibit multifunctional properties due to their structural flexibility, susceptibility to cation substitution, and the mixed valence state of Mn ions. Their physicochemical behavior directly depends on the symmetry of the crystal structure, the distortion of MnO_6_ octahedra, the Mn–O–Mn bond angle, oxygen stoichiometry, and nanoscale effects. These factors allow for effective control of magnetic properties, electrical conductivity, dielectric response, optical absorption, and catalytic activity.

As outlined in the review, the correct choice of synthesis method determines the phase purity, particle size, morphology, and functional properties of the resulting manganites. Although the traditional solid-state processing method is simple, it is limited by high temperatures and the formation of a coarse-grained product, whereas sol–gel, Pecini, hydrothermal, and other mild chemical approaches provide better homogeneity, low-temperature phase transformation, and control over nanostructures. Therefore, optimizing synthesis conditions depending on the specific application is one of the main ways to improve the material’s performance.

Perovskite manganites hold significant potential in fields such as magnetic refrigeration, spintronics, sensing, photocatalysis, heterogeneous catalysis, electrocataly sis, supercapacitors, batteries, and solid-oxide fuel cells. The ability to improve their magnetocaloric, electrical, and catalytic properties, particularly through doping of A- and B-sites, control of oxygen vacancies, and interface engineering, makes these materials particularly important from an applied perspective.

## 6. Conclusions

This review study provides a comprehensive analysis of current scientific research on the synthesis, classification, structural characteristics, and applications of perovskite manganites. A systematic review of the literature revealed that the properties of these materials are closely related to their crystal structure, Thorus distortions (Jahn–Teller effect), oxygen non-stoichiometry, and cation doping.

Results of the analysis showed that perovskite manganites, as multifunctional materials, possess high scientific and practical potential. Particular interest has been shown in recent years in their magnetocaloric and magnetoresistive properties, as well as their electrocatalytic activity (in HER, OER, and ORRs), photocatalytic and electronegative characteristics. This makes them suitable for a wide range of applications in energy, environmental science, and sensor technologies. It has been demonstrated that structural modifications—specifically doping, defect control, and nanostructuring—play a crucial role in controlling the functional properties of these materials. This research enables the enhancement of perovskite manganites’ performance and the expansion of their application scope. Furthermore, the broad range of applications for teldan—which over the past decade has served as a semiconductor, energy converter, and multifunctional material in catalytic and photocatalytic processes—has been highlighted.

Further research should focus on gaining a deeper understanding of the relationship between the structure and properties of perovskite manganites, as well as on improving their stability and efficiency in electrocatalytic and energy applications.

## Figures and Tables

**Table 1:** Classification of synthesis methods for perovskite manganites.

Synthesis Method	Specific Material Examples	Key Characteristics & Conditions
Conventional Solid-State Reaction (SSR) [40,41,42]	YMnO_3_, ErMnO_3_, YbMnO_3_, Sm_1__−x_Sr_x_MnO_3_, Pr_0.6_Sr_0.4_MnO_3_	Requires high temperatures (typically 1000 °C to 1400 °C). Involves mechanical mixing/grinding of oxide/carbonate precursors followed by sintering. Often results in large particle sizes and low surface area [47,48,49,50].
Sol–Gel (Citrate/Polymeric)	NdMnO_3_, LaMnO_3_, La_1−x_Ba_x_MnO_3_	Uses chelating agents (citric acid) and polymerization agents (ethylene glycol). Offers homogeneous blending at the molecular level, high reactivity, and lower phase formation temperatures compared to SSR [51,52,53].
Combustion/Auto-Combustion	LaMnO_3_ nanoparticles, LaMn_1−y_Ni_y_O_3_, La_0.9_A_0.1_MnO_3+δ_ (A = alkali)	Very fast and inexpensive route using organic fuels like oxalic acid, glycine, or polyvinyl alcohol (PVA) with metal nitrates. Produces highly pure, porous, and homogeneous nanograined powders [54,55,56,57].
Pechini Method	La_0.5_Nd_0.2_Ca_0.3__−x_K_x_MnO_3_, LSMN, LSCN	Specific variant of citrate complexation that forms a polymeric resin network. Excellent for maintaining stoichiometry in complex doped systems [48,57,58,59,60,61]
Hydrothermal Method	La_1__−x_KxMnO_3_ microcubes, SrMnO_3_, LSMO, FeMnO_3_ nanoparticles	Conducted in high-pressure reactors under autogenous pressure. Effective for producing single-crystalline nanostructures (like microcubes or nanorods) with controlled morphology using mineralizers (KOH) [48,62]
Molten Method	LaMnO_3_ nanocubes, REMnO_3_ (RE = Y, Er, Tm, Yb)	Synthesis in a flux of melted salts (NaCl or KCl). Useful for preparing micro-sized, hexagonally shaped or nanocubic particles [47,62]
Microwave-Assisted Synthesis	La_0.7_Ba_0.3_MnO_3_ nanoparticles, YMnO_3_	Provides facile, fast, and uniform heating. Significantly reduces reaction times and can be combined with hydrothermal or combustion routes [63,64].
Ultrasonic (Sonochemical)	SrMnO_3_, Pd-CeMnO_3_, BaMnO_3_	Uses ultrasonic waves to induce chemical reactions. It is convenient and cost-effective, often used for preparing catalysts for oxygen evolution [48].
Physical Deposition (PLD/Sputtering)	LaMnO_3_ thin films, LCMO/YBCO heterostructures	Techniques like Pulsed Laser Deposition (PLD) or RF magnetron sputtering are used to grow high-quality epitaxial thin films and superlattices [55,65].
Mechanochemical Synthesis	PrBaMn_2_O_5__+__δ_, BiMnO_3_	Uses mechanical energy (high-energy ball milling) to induce reactions. Efficient for preventing secondary phases and producing nano-range grains [66,67,68].
PVA-Gel Route	Nd_0.67_A_0.33_MnO_3_ (A = Ca, Sr, Pb, Ba)	Precursors are dispersed in PVA, leading to a dry, fluffy, and porous mass that is subsequently burnt and sintered [69].

**Table 2:** Properties and crystal structures of perovskite manganites.

Perovskite Manganite	Crystal Structure (Space Group)	Properties and Applications
LaMnO_3_	*Rhombohedral (R* $\bar{3}$ *c)*	At low temperatures, the material exhibits antiferromagnetic properties. However, ferromagnetic behavior may arise due to the mixed valence of Mn^3+^/Mn^4+^ ions; in this case, the saturation magnetization (M_S_) increases as the annealing temperature rises [55].
HoMnO_3_	*Cubic (Pm3m)* *Hexagonal (P6_3_cm)* *Orthorhombic (Pnma)*	It is most stable in the hexagonal and orthorhombic phases; possible magnetic configurations include NM (non-magnetic state), FM (ferromagnetic), and AFM (antiferromagnetic). In the hexagonal phase, the material exhibits brittle properties, whereas in the cubic phase, it is more ductile. The calculated bulk compression modulus is approximately 154–189 GPa, depending on the crystalline phase [79].
BaMnO_3_	*Hexagonal (P6_3_/mmc)*	Nanorods exhibit extreme sensitivity to gases; used as a catalyst for oxygen evolution during water splitting [11].
SrMnO_3_	*Hexagonal (P6_3_/mmc)*	G-type antiferromagnetic insulator; exhibits metallic character at the Fermi level via DFT [48].
SmMnO_3_	*Orthorhombic (Pbnm)*	Pure form is A-type AFM (*T_N_* = 56 K); Sr-doping increases *TN* to 90 K and significantly boosts saturation magnetic moment [50].
YMnO_3_	*Hexagonal (P6_3_cm)*	Paramagnetic to antiferromagnetic (AFM) transition at low temperatures; Néel temperature (T_N_) increases as ionic radius decreases (73.6 K for Y to 87.3 K for Yb). Narrow optical band gaps (1.62–1.64 eV); exhibit leaky ferroelectric behavior and frequency-dependent dielectric constants [49].
ErMnO_3_		
YbMnO_3_		
NdMnO_3_	*Orthorhombic (Pbnm)*	Multiferroic at room temperature; exhibits Jahn–Teller distortion; used in piezoelectric nanogenerators [52].
PrMnO_3_	*Orthorhombic (Pnma)*	Half-metallic ferromagnetic; high Young’s modulus (mechanical strength); TC transition observed around 485 K (Debye temperature) [86].
CeMnO_3_	*Cubic (Fm-3m)*	Degradation of antibiotics and dyes under visible light; Photocatalytically active structure; high electrical conductivity; good electrochemical performance; promising Li-ion battery anode material [87,88].
La_0.6_MnO_3_	*Orthorhombic*	A-site deficient; exhibits high density of oxygen vacancies; retains catalytic activity for formaldehyde oxidation for 63 h [89].
La_0.73_K_0.27_MnO_3_	*Rhombohedral (R* $\bar{3}$ *c)*	Synthesized as microcubes; exhibits 98% conversion efficiency in the reduction of α,β-unsaturated carbonyl compounds [62].
La_0.9_Rb_0.1_MnO_3+δ_	*Rhombohedral (R* $\bar{3}$ *c)*	Single-phase catalyst for soot oxidation; unit cell volume increases with the ionic radius of the alkali dopant [54].
Nd_0.7_Ca_0.3_MnO_3_	*Orthorhombic (Pnma)*	Average crystallite size 18 nm; shows cluster glass-like state with ferromagnetic/antiferromagnetic coexistence; TC = 33 K [90].
Nd_0.67_Pb_0.33_MnO_3_	*Orthorhombic (Pnma)*	Ferromagnetic ordering with Curie temperature 165 K [69].
La_0.7_Ba_0.3_MnO_3_	*Rhombohedral (R* $\bar{3}$ *c)*	Semiconductor nature with tunable bandgap; used for photocatalytic degradation of methyl orange [63].
La_0.7_Sr_0.3_MnO_3_	*Rhombohedral (R3c)*	Half-metallic with high spin polarization; TC = 369 K; extensively studied for spintronics and biomedical MRI applications [91].
La_0.85_Mg_0.15_MnO_3_	*Rhombohedral (R* $\bar{3}$ *)*	Pure rhombohedral phase formed at x < 0.15; Mg doping reduces T_C_ and induces lattice tilting [91].
LaMn_0.6_Ni_0.4_O_3_	*Rhombohedral*	Optimized for spintronics with high saturation magnetization (Ms = 53.27 emu/g); T_C_ tuned to 246 K [57].
La_0.85_Fe_0.7_Mn_0.3_O_3_	*Orthorhombic*	Ferromagnetic with collective magnetism; shows phase heterogeneity in ESR studies; sensitive redox behavior in H_2_ atmospheres [92].
LaCu_0.3_Mn_0.7_O_3_	*Orthorhombic (Pbnm)*	Copper-substituted manganite; higher Cu levels (up to 50%) maintain the structure, but 70%+ Cu leads to secondary phases (CuO, La_2_CuO_4_) [93].
LaCu_0.5_Mn_0.5_O_3_	*Orthorhombic (Pbnm)*	Replacing manganese with copper enhances catalytic activity in oxidation reactions due to interfacial synergy [93,94].
Ba_0.8_Mn_0.7_Cu_0.3_O_3_	*Hexagonal*	Non-stoichiometric catalyst for CO oxidation; Ba deficiency promotes the formation of active oxygen vacancies [95].
Ca_0.82_La_0.18_MnO_3_	*Orthorhombic*	Electron-doped manganite; the nanoparticles exhibit ferromagnetism (Tc = 165 K), while the nanowires remain in an antiferromagnetic state [11].
La_0.7_Sr_0.3_Mn_0.8_Fe_0.2_O_3_	*Rhombohedral*	Iron doping allows for the tuning of the magnetocaloric effect and the Curie temperature in magnetic cooling systems [16].
Sm_1−x_Sr_x_MnO_3_	*Orthorhombic (Pbnm)*	T_N_ increases with Sr doping (90 K at x = 0.25); used for solar energy capture and CO_2_ conversion [50].
Bi_1−x_Sr_x_MnO_3_	*Tetragonal/Orthorhombic*	Discovers a new type of charge ordering (“Mn^3+^ stripes”) at room temperature [96].
La_0.9_Li_0.1_MnO_3+δ_	*Rhombohedral (R* $\bar{3}$ *c)*	Highest specific surface area (20.1 m^2^/g) in its series; highly active in catalytic soot oxidation [54].
La_0.5_Nd_0.2_Ca_0.25_K_0.05_MnO_3_ (LCKM)	*Orthorhombic (Pbnm)*	Exhibits mixed valence (Mn^3+^/Mn^4+^); high regenerative capacity when used as a catalyst [59].
La_0.8_Ca_0.15_Na_0.05_Mn_0.8_Fe_0.2_O_3_	*Orthorhombic (Pnma)*	Curie temperature T_C_ = 111 K [97].
La_0.7_Ca_0.3_Mn_1−x_Ni_x/2_Ti_x/2_O_3_	*Orthorhombic (Pnma)*	Effective microwave absorber; provides up to 98% absorption at 8.24 GHz [98].
La(Ti_0.2_Mn_0.2_Fe_0.2_Co_0.2_Ni_0.2_)O_3_	*Hexagonal*	High-entropy perovskite; effective in dry reforming of ethanol; exhibits moderate oxygen mobility [99].
EuTi_0.8_Nb_0.1_Mn_0.1_O_3_	*Cubic*	Complex substitution in the B-site; studied for the control of magnetocaloric properties at ultra-low temperatures [16].
CeBaMn_2_O_6_	*Layered Orthorhombic (Pnma)*	Exhibits magnetoelectric nanoregions; largest reported size variance for an A-site substituted perovskite [100].
Gd_2_NiMnO_6_	*Double Perovskite/monoclinic (P2_1_/n)*	High electrochemical activity with pseudocapacitive behavior; specific capacitance 400 F g^−1^, material for energy storage applications [101].
Pr_2_NiMnO_6_	*Monoclinic*	T_C_ = 215 K; studied for high-efficiency magnetic refrigeration technology (MRT) [16].
Nd_2_NiMnO_6_	*Monoclinic*	T_C_ = 191 K; exhibits ferromagnetic ordering due to superexchange interactions between Ni^2+^ and Mn^4+^ [16].
La_2_CoMnO_6_	*Cation-ordered*	Spontaneous cationic ordering observed in thin films; studied for magnetoelectric and spintronic applications [102].

## Data Availability

No new data were created or analyzed in this study. Data sharing is not applicable to this article.
